# Monitoring Campaign over an Edible Dormouse Population (*Glis glis*; Rodentia: Gliridae) in Sicily: First Report of Mesocestodiasis

**DOI:** 10.3390/ani11040934

**Published:** 2021-03-25

**Authors:** Giorgia Schirò, Domenico Pieri, Mario Lo Valvo, Luigi Gradoni, Simone Mario Cacciò, Francesco Severini, Gianluca Marucci, Lucia Galuppo, Valentina Cumbo, Roberto Puleio, Guido Ruggero Loria

**Affiliations:** 1 Istituto Zooprofilattico Sperimentale della Sicilia, Via Gino Marinuzzi 3, 90129 Palermo, Italy; galuppolucia@gmail.com (L.G.); valentina.cumbo@gmail.com (V.C.); roberto.puleio@izssicilia.it (R.P.); guidoruggero.loria@izssicilia.it (G.R.L.); 2 STEBICEF, Università degli Studi di Palermo, Via Archirafi, 18, 90123 Palermo, Italy; dome.pieri89@gmail.com (D.P.); mario.lovalvo@unipa.it (M.L.V.); 3 Istituto Superiore di Sanità, Viale Regina Elena 299, 00161 Rome, Italy; luigi.gradoni@iss.it (L.G.); simone.caccio@iss.it (S.M.C.); francesco.severini@iss.it (F.S.); gianluca.marucci@iss.it (G.M.)

**Keywords:** dormice, *Glis glis*, *Monopsyllus sciorum*, *Mesocestoides lineatus*

## Abstract

**Simple Summary:**

In Nebrodi Park (Sicily, Italy), live many wild mammal species that move closer to human beings every day. The edible dormouse (*Glis glis*), in 2017 and 2018, was responsible for nut crop damage in the area. For this reason, a sanitary monitoring campaign involving 30 dormice was carried out by collecting rectal and conjunctival swabs and fur and nest content, which were then processed for laboratory examinations. A large presence of fleas belonging to *Monopsyllus sciurorum* was found. Necropsy of a dead dormouse revealed an infection of *Mesocestoides lineatus*, whose cysts were found in the abdomen cavity and on the liver; this is the first report of this in this species. Further studies are necessary to identify their role in the environment, considering the limited knowledge of this species in Italy.

**Abstract:**

This study reports on the health status of the edible dormouse (*Glis glis*) living in Nebrodi Park (Sicily, Italy), responsible for nut crop damage in the area. In the frame of a monitoring campaign for potential zoonotic risk involving 30 dormice, rectal and conjunctival swabs and fur and nest content were collected for bacteriological and parasitological examinations, respectively. A large presence of fleas belonging to *Monopsyllus sciurorum* was found. Necropsy of a dead dormouse revealed an infection of *Mesocestoides lineatus*, whose cysts were found in the abdomen cavity and on the liver; this is the first report of this in this species. Further studies are necessary to identify their role in the environment, considering the limited knowledge of this species in Italy.

## 1. Introduction

This study was carried out in a Sicilian regional park, located in the Nebrodi mountains (Sicily, Italy), which still represents a sanctuary for biodiversity, hosting several animal species including a variety of micromammals. The edible dormouse (*Glis glis* L.; Rodentia: Gliridae), an arboreal hibernating rodent, is one of the inhabitants of this rare Mediterranean landscape, still reported in the central–eastern areas of Sicily, including Madonie, Nebrodi, Peloritani, the Iblei mountains, and Mount Etna. This species usually lives in arboreal nests, which are generally dug by other animals in the hollows of trees, and its mobility in the environment is both vertical and horizontal [1]. Reproduction of the dormouse is directly related to food availability, generally nuts from beech and oak trees [2,3,4]; mating, and consequently the lifespan of these animals, is related to the intensity and frequency of mast seeding events [5]. The site fidelity of this species is well known [6,7,8]; nevertheless, they may move to look for better nesting sites and, moreover, availability of food resources [9].

Although little is known about the sanitary state of edible dormice in Europe, this species may act as a reservoir host for zoonotic diseases, especially those transmitted by fleas and ticks [1], but also for emerging viral disease, such as hantavirus, monkeypox, and polyomaviruses [10,11,12]. In particular, edible dormice seem to be, more than any other mammals, an efficient reservoir host for Lyme disease spirochetes (*Borrelia* spp.) transmitted by ticks belonging to the Ixodidae family, such as *Ixodes ricinus* [13,14]. The prevalence of the infection in this species is probably due to its longevity (average 9 years) and to the numerous ticks that feed on them [15]. Moreover, wild rodents are an important source of the transmission of *Francisella tularensis*, which in the last few years has been considered as a re-emerging zoonosis [16,17].

In the last decade, a massive increase in the dormouse population living in the Nebrodi mountains has been the source of worries and target of protests by local farmers because it was believed to be responsible for huge losses of their hazelnut crop, one of the most appreciated and valuable products in these areas. Therefore, between 2017 and 2018, the Sicilian region, together with University and Public Veterinary institutions officially involved in management and control of the risks caused by regional fauna, implemented a survey to monitor the size of the dormouse population and to evaluate the losses due to nut crop damage. In order to monitor the range of migration of the rodents in the following months, all trapped animals were identified by the application of a microchip.

The surveillance activities aimed to understand if the cause of the decrease in the hazelnut crops were related to the increased dormice population, and to evaluate the eventual relocation of this species. For these reasons, the authors sampled several rodents to monitor their health status, reporting some interesting data on pathogens affecting dormice living in this area.

## 2. Materials and Methods

### 2.1. Sample Collection

**Figure 1:**
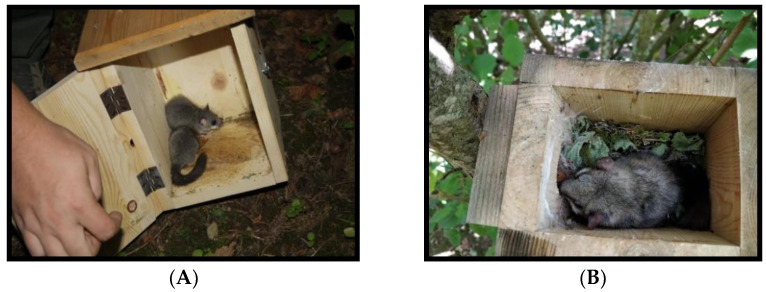
(**A**) Dormice trapped in a wooden nest; (**B**) Dormouse found dead in a nest.

A trapping campaign was carried out from October 2017 to October 2018; a total of 30 dormice were trapped in artificial wooden nests utilized to monitor the presence and density of the species in the area (Figure 1A); once identified by a microchip implanted subcutaneously in the neck, rectal and conjunctival swabs were immediately obtained while searching for ectoparasites on the rodents, which were released thereafter. Furthermore, in 2018, one carcass of a male adult dormouse was also found in a nest. The rodent was in a somewhat lethargic position, as it probably passed away while sleeping. (Figure 1B).

### 2.2. Parasitological Investigations

Dormice fur and the content of their nests were carefully inspected for any ectoparasites with the help of disposable gloves, a brush, and surgical forceps. At the laboratory, collected specimens were fixed in 70% (*v*/*v*) ethanol for morphological identification. Fleas were identified by a comparison of dichotomous keys according to standard taxonomic keys [18]. The round-shaped endoparasites detected in the abdomen of the dormouse carcass were also fixed in 70% (*v*/*v*) ethanol in order to evaluate characteristic features and morphology using the classification approaches for Nematoda and Cestoda. To confirm the morphological identification, the sample was also tested by a PCR protocol, amplifying a fragment of gene coding for cytochrome oxidase I subunit (COI). A couple of universal primers [19] were used to amplify a variable region of approximately 450 bp [20] in the mitochondrial cytochrome c oxidase I (COI) gene. The used primers were JB3 (5′ TTTTTTGGGCATCCTGAGGTTTAT 3′) and JB4.5 (5′ TAAAGAAAGAACATAATGAAAATG 3′).

### 2.3. Investigation for Tularemia

Fleas were also screened for DNA belonging to *Francisella tularensis* by real-time PCR [21]. The insects were firstly washed three times in 70% ethanol, rinsed in sterile distilled water, and left to dry at room temperature. They were then homogenized by mechanical agitation, and total DNA was purified using a DNeasy Blood and Tissue Kit (Qiagen, Milan, Italy) according to the manufacturer’s instructions. Purified DNA was eluted in a 200 μL AE (Elution) buffer. Detection of *Francisella tularensis* DNA was performed in a CFX-96 real-time system (Biorad, Milan, Italy) by real-time PCR targeting the 23 kDa gene, as described. Negative (no template) and positive (DNA of *F. tularensis* subsp. *tularensis* strain ATCC 6223) controls were included in each run, and an internal control was included in each sample (Taqman Exogenous Internal Positive Control, AB Applied Biosystems).

### 2.4. Bacterial Isolation

From each live rodent, rectal and conjunctival swabs were collected for bacterial investigation. Rectal swabs were cultured on Columbia and McConkey agars and for Gram-negative bacteria; both were incubated aerobically at 37 °C for 24 h (up to 48). For the isolation of *Salmonella* spp., a pre-enrichment was firstly conducted by soaking the swab in 10 mL of Buffered Peptone Water (APW) at 37 ± 1 °C for 16–20 h; then, 1 mL and 100 µL of APW were respectively transferred in to 9 mL of Selenite Cystine Broth (24 h at 37 °C) and in to 10 mL of Rappaport Vassiliadis Broth (24–48 h at 42 °C). Broth cultures were plated in a selective media Xylose Lysine Deoxycholate (XLD) agar and Brilliant Green (BGA) agar, incubated at 37 °C for 24 h.

For the isolation of *Mycoplasma* spp., conjunctival swabs were sub-cultured in a *Mycoplasma* broth base with supplement G (Oxoid Limited, Hampshire, UK) and sterile equine serum and incubated at 37 °C in an atmosphere containing 10% CO_2_ for 4 to 7 days. After incubation, with a sterile calibrated loop (10 µL) and an aliquot of *Mycoplasma* broth, cultures were transferred onto a Mycoplasma agar and incubated under identical conditions. The plates were examined daily with a stereomicroscope until up to a maximum of 25 days.

The identification of isolates was carried out with biochemical and enzymatic assays starting from cloned colonies, obtained from a brain heart infusion (BHI) agar. Isolated strains were subjected to various tests: catalase and oxidase assays, Gram staining, urea, indole, citrate, and mobility tests. Sugars, such as sorbitol, sucrose, lactose, and malonate [22], were also tested. For other Gram-positive strains, a commercial kit from API NE^®^ (bioMérieux, Bagno a Ripoli, Italy) systems was used.

### 2.5. Anatomopathological and Histological Examination

The dormouse found dead was subjected to necropsy, during which, tissue samples were collected from all suspected lesions and fixed in 10% neutral buffered formalin. Tissues were processed by routine paraffin embedding and 4-μm-thick sections were mounted and stained with hematoxylin and eosin (HE).

## 3. Results

Among 30 dormice trapped alive, 21 males and 9 females (14 young aged under 5 months and 16 adults) were identified. All rodents were apparently healthy and reactive during handling procedures for the implantation of the subcutaneous microchip. Some of them (no. 4) showed a massive presence of fleas on their fur. Fleas were also infesting the material collected in the artificial nests. No other species of ectoparasites were found in this group of dormice nor in their nests.

**Figure 2:**
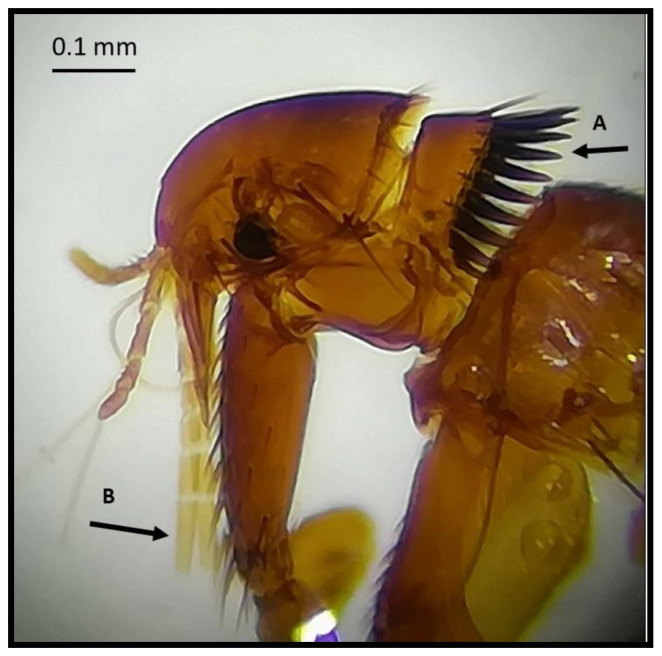
(**A**) Pronotal comb consisting of less than 24 spines; (**B**) labial palp consisting of at least 4 segments and reaching the anterior trochanter as much as possible.

**Figure 3:**
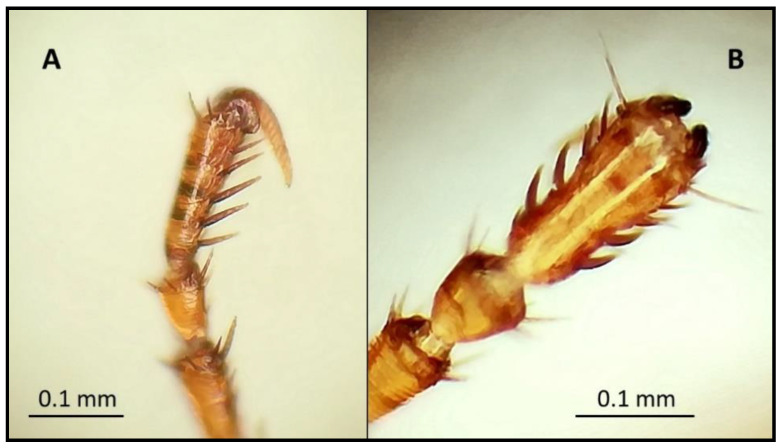
Last article of all tarsi with 5 pairs of lateral plantar bristles (**A**) side view and (**B**) front view).

**Figure 4:**
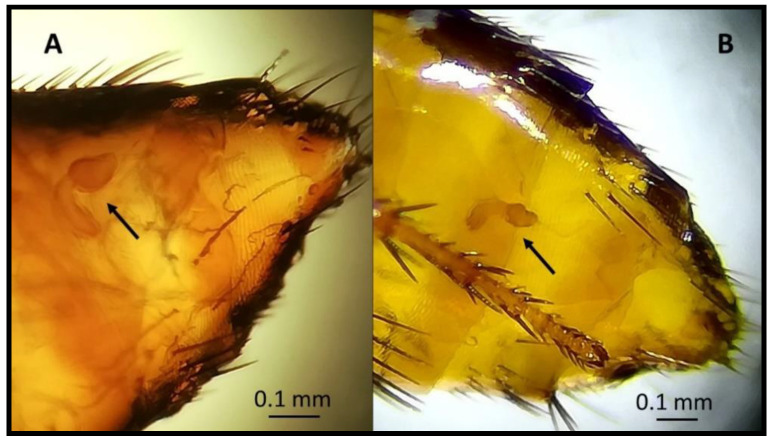
Head (**A**) of the spermatheca as long as the tail (**B**) (and tail without papilla).

During the morphological examination, all flea specimens collected were found to belong to the species *Monopsyllus sciurorum*. The main characteristic of the species identification was the spermatheca’s morphology, whose head appeared pyriform, and the copulatory bag was not spiral-shaped (Figure 2, Figure 3 and Figure 4). In detail, the set of characteristics leading to species identification in our specimens belonged to the following three groups:

(1) Characteristics related to superfamilies and families:
  Posterior hip without small spiny bristles on the underside of the inner side;
  Wipe abdominals, each with more than one row of bristles (characteristic of the Ceratophylloidea superfamily);
  Of the two apical bristles of the anterior femur, the external one is longer;
  Methane with small marginal spines.
(2) Characteristics of the genus:
  Comb of the pronotum consisting of less than 24 spines;
  Labial palp consisting of at least four segments and reaching the anterior trochanter as much as possible;
  Last item of all tarsi with five pairs of lateral plantar bristles;
  Head of the spermatheca as long as the tail (and a tail without papilla).
(3) Characteristics of the spermatheca:
  Spermatheca’s head is piriform, and the copulating bag is non-spiral shaped: *Monopsyllus sciurorum*;
  Spermatheca’s head is globular and shows a spiral-shaped copulating bag: other species of *Monopsyllus* genus.

Molecular investigation carried out on these insects at the National Reference Laboratory for Tularemia (Istituto Zooprofilattico Sperimentale della Lombardia edEmilia Romagna) did not detect any positive samples for *F. tularensis*.

Fourteen out of 30 rectal swabs tested positive for bacterial isolation, from which 16 Gram-negative bacterial strains were identified. Thirteen belonging to the Enterobacteriaceae family, such as *E. coli* (no. 10, 33.33%), *Klebsiella oxytoca* (no. 2, 6.66%), *Enterobacter* spp. (no. 1, 3.33%); the other isolated strains were *Pseudomonas luteola* (no. 1, 3.33%), *Aeromonas hydrophila* (no. 1, 3.33%), and *Pantoea* spp (no. 1, 3.33%). All samples tested negative for *Salmonella* spp., and all conjunctival swabs tested negative for *Mycoplasma* spp. and other pathogenic bacteria.

**Figure 5:**
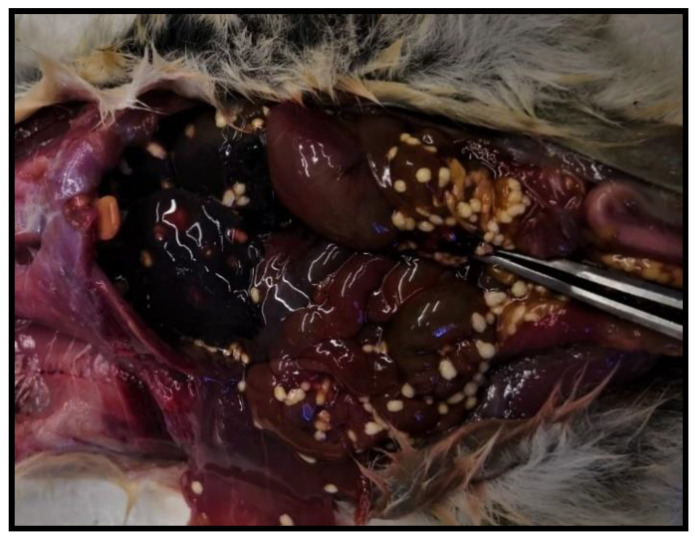
Parasite cysts in peritoneal cavity and on liver.

Necropsy of the dead dormouse highlighted its poor body condition; serosanguineous fluid was observed in the abdominal cavity along with severe liver congestion. Moreover, in the peritoneal cavity and above the surface of the liver, multiple small, whitish, cyst-like bodies (2–3 mm) were visible (Figure 5). 

**Figure 6:**
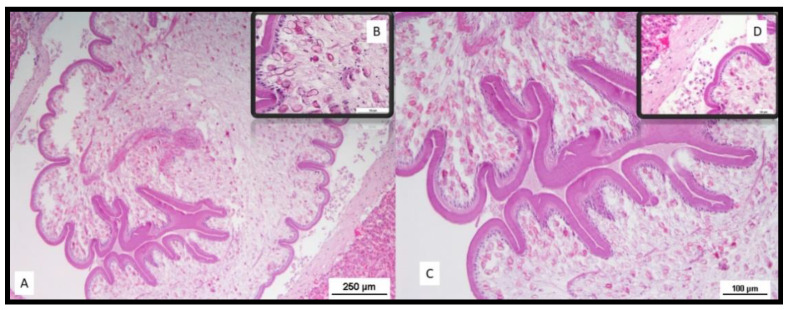
Histological lesions; (A) microscopic picture of *Mesocestoides lineatus* cysts on liver, hematoxylin and eosin (HE) stain. Bar = 250 µm; (B) inset: microtriches, which are fine hair-like projections of the cuticle, features typical of cestodes; (C) tetrathyridium with scolex withdrawn into a deep invagination canal. Bar = 100 µm; (D) inset: tegument surrounded by a loose connective tissue stroma and some inflammatory cells.

Sections from the portions of tissue presenting suspected nodules/bodies within the liver parenchyma were sampled and fixed in 10% neutral-buffered formalin. Histological examination of liver sections showed a parasitic stage known as tetrathyridia, surrounded by a loose connective tissue stroma and some inflammatory cells (Figure 6). Tetrathyridia were elongated with a pointed posterior end and had an invaginated scolex at the anterior wider portion, longer than wide (1200–2360 by 430–890 µm in size). Thin sections of tetrathyridia showed a 15–30 μm thick tegument with an underlying basal matrix covering the tetrathyridium and the canal leading to the anterior end with the invaginated scolex (Figure 6). A circular muscle layer divided the parenchyma into an inner medullary zone and an outer cortical zone. Longitudinal muscles were observed underneath the tegument layer. Moreover, features typical of cestodes were also visible, such as the microtriches, which are fine hair-like projections of the cuticle.

A laboratory investigation carried out at the Department of Infectious Diseases, Reference Laboratory for Parasites of Europe (Istituto Superiore di Sanità, Italy) on several larval stages collected from the abdominal cavity of the dormouse confirmed the phenotypic classification by PCR and sequencing of cytochrome c oxidase subunit I (COI) as the tapeworm *Mesocestoides lineatus* (Cyclophyllidea: Mesocestoididae).

## 4. Discussion

This monitoring campaign reported the first veterinary study of dormice in Sicily. Several studies have reported ecological and life cycle data of dormice [2,5,7], but little is known about their health status and diseases. Moreover, these studies were unrelated to the European population.

Diseases of dormice are rarely reported, and the knowledge of the pathogens this species is at risk of is very limited. The few reports available in the EU concern parasitic, bacterial, and viral diseases. The ecology of the dormouse, a rodent sharing its nest with many other animals, is likely to facilitate the exchange of parasites and other pathogens. This is the probable reason why dormice in the EU have been found to host 29 different species of fleas—only a few are specific to the species, with many other parasites coming from other hosts [1]: some species of Hymenolepididae (Cestoda) [23]; other species belonging to Nematoda phylum, such as *Pseudophysaloptera kahmanni*, *Gongylonema pithyusensis*, and *Heligmosomoides polygrus* [24,25,26]; and zoonotic microbial diseases, such as tularemia [27] or hantaviruses, which may present a potential risk for human diseases by direct contact [10].

Dormice, as with other rodents, are recognized reservoir hosts of zoonotic diseases and, in particular, those transmitted by vectors like fleas and ticks [1]. Despite the 29 different species of fleas recognized to infest dormice across Europe [1,28,29], *Monopsyllus sciurorum* was the only species detected on the dormice of the Nebrodi mountains. Of note, this genus had been previously identified in Sicily on the dormice trapped in the woodlands of Biviere lake, the Cesarò area, in the Messina province [30], and was also mentioned in previous reports [31].

Fleas could be responsible for the transmission of zoonotic pathogens, such as *Yersinia* spp. and *Francisella* spp. [32,33]; moreover, flea parasitism can cause blood loss and skin damage, influencing the welfare of dormice.

The swabs and the sampling of the ectoparasites allowed an assessment of the sanitary state of this species that often shares its environment with other wild and domestic animals and human beings. The analyses on the bacterial isolation in this dormouse population indicated that most of the bacterial species isolated from rectal swabs belong to the *Enterobacteriaceae* family, and in particular, the main strain isolated was *E. coli*, which suggests that this rodent species is probably not involved in shedding through fecal bacteria like *Salmonella* spp., as with other wild animals [34]. 

This study reports the first evidence of infection by *Mesocestoides* spp. (Cestoda) in *G. glis*. Cyclophyllidea tapeworms belonging to the genus *Mesocestoides* are commonly detected in wild rodents, and the adult stage of this parasite affects several species of domestic and wild carnivores, which represent the final host. *Mesocestoides* spp. spread their eggs to the environment within the proglottids; *M. lineatus* occurs in cats, dogs, and foxes. The adult parasites reach a length of about 30–80 cm and a width of about 2–3 mm, and their mouth is composed of a scolex of medium size with four suckers without hooks.

Larval stages of this parasite, particularly the second larval stage, the tetrathyridium, develop in the peritoneal cavity and musculature of several species belonging to different taxonomic groups, including amphibians, reptiles, birds, and mammals (including cats). Some infestations can contain large numbers of larvae.

Current studies suggest that the cycle involves three hosts: an arthropod as an intermediate host, a vertebrate intermediate host (several taxonomic groups including rodents), and a carnivore definitive host, but part of the life cycle of *Mesocestoides* spp. remains unknown. Several arthropods have been investigated as first intermediate hosts, including ants and oribatid mites, without success. However, the wide distribution of the parasite should concern very common species of Arthropoda; moreover, infections caused by different stages of *Mesocestoides lineatus* have been reported in many countries in Europe, Asia, and Africa [35].

Humans can be an accidental host, but the infection is usually linked to the ingestion of raw viscera contaminated by infective metacestode larvae; human infections have been reported in Korea, Japan, China, and the USA [36,37,38,39].

Despite the fact that rodents are long known to be part of the *Mesocestoides* life cycle as second intermediate hosts, the edible dormouse was never reported to be infected by this parasite. Indeed, the possibility to relocate this population to limit nut crop damage is strictly linked to their health status.

## 5. Conclusions

This study presents a contribution to the knowledge of diseases of the *G. glis*, which is considered as endangered in some areas of Europe and is subject to repopulation programs, such as that reported in Lithuania [1]. The monitoring activities on the dormouse population allowed the evaluation of their health status and associated potential zoonotic pathogens, considering the limited knowledge of this species in Italy. Further investigations are necessary to identify the ecological role of the edible dormice in the environment and also its impact on agriculture activities that put its future conservation at risk.
